# Ubiquitin-Proteasome-Mediated Protein Degradation and Disorders of the Central Nervous System

**DOI:** 10.3390/ijms26030966

**Published:** 2025-01-24

**Authors:** Ashok N. Hegde, Logan E. Timm, Connor J. Sivley, Shrenik Ramiyaramcharankarthic, Olivia J. Lowrimore, Brenna J. Hendrix, Teodora G. Grozdanov, William J. Anderson

**Affiliations:** Department of Biological and Environmental Sciences, Georgia College and State University, Milledgeville, GA 31061, USA; logan.timm@bobcats.gcsu.edu (L.E.T.); connor.sivley@bobcats.gcsu.edu (C.J.S.); shrenik.ramiyaramcharankarthi@bobcats.gcsu.edu (S.R.); olivia.lowrimore@bobcats.gcsu.edu (O.J.L.); brenna.hendrix@bobcats.gcsu.edu (B.J.H.); teodora.grozdanov@bobcats.gcsu.edu (T.G.G.); william.anderson1@bobcats.gcsu.edu (W.J.A.)

**Keywords:** proteolysis, mental disorders, neurodegenerative, pathology, E3 ligases, deubiquitinating

## Abstract

Ubiquitin–proteasome-mediated proteolysis post-translationally regulates the amounts of many proteins that are critical for the normal physiology of the central nervous system. Research carried out over the last several years has revealed a role for components of the ubiquitin–proteasome pathway (UPP) in many neurodegenerative diseases such as Parkinson’s disease and Huntington’s disease. Studies have also shown a role for the UPP in mental disorders such as schizophrenia and autism. Even though dysregulation of protein degradation by the UPP is a contributory factor to the pathology underlying many nervous system disorders, the association between the components of the UPP and these diseases is far from simple. In this review, we discuss the connections between the UPP and some of the major mental disorders and neurodegenerative diseases.

## 1. Introduction

Advancing the knowledge of the normal workings of the central nervous system (CNS) as well as its diseases and disorders can be greatly aided by a mechanistic understanding of molecular pathways. Over the years, much has been learned regarding the synthesis of macromolecules, followed by discoveries regarding the roles of protein degradation. Specifically, ubiquitin–proteasome-mediated proteolysis has been shown to function in regulating many physiological processes in the body [1,2], including those of the CNS.

Many studies also have implicated ubiquitin-mediated protein degradation in the development of maladies such as cancer [3] as well as diseases of the nervous system [4]. Even before the discovery of its role in normal cellular physiology, ubiquitin was found to be associated with brain pathology [5], leading to the idea that ubiquitin is important for the degradation of abnormal proteins. Subsequently, investigations on the degradation of cell cycle-regulating proteins [6] suggested that ubiquitin–proteasome-mediated degradation plays a critical role in post-translationally managing the amounts of proteins available in the cell. Detailed understanding of ubiquitin–proteasome-mediated degradation has resulted in insights into disease processes such as cancer. For example, the tumor suppressor protein BRCA1 is a ligase that ubiquitinates cyclin B and Cdc25C, which are mitotic cyclins critical for the G2/M transition [7]. Therefore, mutations in BRCA1 that hinder this function prime the cell for tumor formation. A second example is that of Cockayne Syndrome (CS) (named after English physician Edward Cockayne). CS is a neurodevelopmental disorder caused by mutations in three types of proteins: CSA, CAB, and CSC. It has been shown that CSA localizes to the centrosome in the prometaphase and metaphase stages and attaches ubiquitin to cyclin B1. The lack of this function causes caspace-3 activation and apoptosis, which are likely to contribute to the development of CS [8]. Subsequent to the early studies on the role of protein degradation in the cell cycle, regulated proteolysis mediated by the ubiquitin–proteasome pathway (UPP) was found to play a role in synaptic plasticity as well as in the development of the nervous system.

Although much of the early research focused on the link between ubiquitin–proteasome-mediated proteolysis and neurodegenerative diseases, later investigations found associations between protein degradation and mental disorders [9,10,11]. It appears that impairment of proteolysis can not only cause overt brain pathology and neurodegeneration, but also abnormalities of nervous system physiology, which manifest as mental disorders [12]. We first describe the components of the UPP and their roles in protein degradation and then describe some of the diseases and disorders of the nervous system with which molecules of the UPP have been associated.

## 2. The Ubiquitin–Proteasome Pathway

The process of proteolysis through the ubiquitin–proteasome pathway (UPP) is highly coordinated. Proteins destined for degradation are marked by covalent attachments of ubiquitin, a small 76-amino-acid protein.

**Figure 1:**
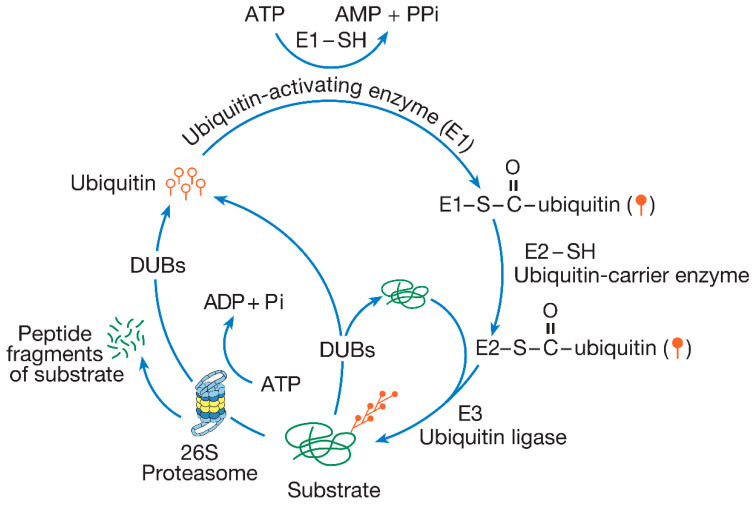
The ubiquitin-proteasome pathway. In this protein degradation pathway, ubiquitin (depicted as a single ubiquitin molecule represented by open circles with straight tails) is specifically and covalently attached to the substrate. The enzymatic process through which ubiquitin is attached to substrates is referred to as ubiquitination or ubiquitin conjugation, which is dependent on the activities of three distinct enzyme classes: E1, E2, and E3. Initially, ubiquitin is activated by E1 to form a ubiquitin–AMP intermediate. This activated ubiquitin (shown as closed circles with straight tails) is then transferred to E2, which serves as a ubiquitin carrier enzyme. E2 subsequently passes the activated ubiquitin to E3, a ubiquitin ligase, which catalyzes the attachment of ubiquitin to the substrate. Additional ubiquitin molecules are then added to the ubiquitin attached to the substrate, leading to the formation of a polyubiquitin chain through successive ubiquitin linkages. Polyubiquitinated substrates are degraded by the 26S proteasome, a proteolytic complex, in an ATP-dependent manner. Although the polyubiquitin chain is disassembled and recycled by deubiquitinating enzymes (DUBs), ubiquitin itself is not degraded. Ubiquitination is reversible prior to degradation by the proteasome, and DUBs can dismantle the polyubiquitin chain if an error in ubiquitination occurs, preventing the substrate from being degraded (Figure modified from Hegde 2004 and reprinted with permission from Elsevier© 2004) [1,2].

The regulation of degradation by the UPP occurs both spatially and temporally. The process of tagging a protein substrate with ubiquitin is carried out by three enzymes: E1, E2, and E3. First, ubiquitin is activated by the enzyme E1. Once activated, ubiquitin is transferred to an enzyme from a family called E2s, which then associates with E3 ubiquitin ligases. The E3 enzymes ligate the ubiquitin molecule to a lysine (Lys) residue on the protein substrate. A second ubiquitin is then covalently attached to the first, forming a polyubiquitin chain. This polyubiquitinated substrate is recognized by the proteasome, which then degrades the protein into smaller peptides and amino acids. The proteasome responsible for degrading polyubiquitinated proteins is known as the 26S proteasome, a name derived from its sedimentation coefficient. Only the protein substrates are degraded, whereas the polyubiquitin chains are disassembled by enzymes called deubiquitinating enzymes (DUBs) and recycled (Figure 1).

The polyubiquitin chain that marks substrates for degradation is typically formed through linkage at Lys-48 of the ubiquitin molecule. However, polyubiquitin chains can also form in other lysine residues in the ubiquitin molecule, such as Lys-6, Lys-11, Lys-27, Lys-29, Lys-33, Lys-48, and Lys-63, and these alternative linkages serve other functions, including signaling for endocytosis [1,13].

### 2.1. Ubiquitin Conjugation

Ubiquitin attachment to a substrate protein occurs through a series of enzymatic steps known as ubiquitin conjugation (ubiquitination). The initial step in this process is the activation of ubiquitin by the enzyme E1, a reaction that requires ATP. Hydrolysis of ATP results in the formation of AMP, which forms a high-energy thiol–ester bond between ubiquitin and AMP. Ubiquitin, thus activated, is then transferred to E2, which is also known as the ubiquitin carrier protein. From E2, the ubiquitin molecule is passed on to E3, which ligates the ubiquitin to the ε-amino group of a lysine residue on the substrate protein. A second ubiquitin molecule is then attached to an internal lysine residue on the first ubiquitin, leading to the formation of a polyubiquitin chain.

### 2.2. Ubiquitin-Conjugating Enzymes: E1, E2, and E3

Among the three classes of ubiquitin-conjugating enzymes, E1 is the least regulated at the physiological level. In contrast, E2 enzymes are more selective, believed to interact with specific E3s. The specificity of the ubiquitin-conjugation reaction is largely determined by E3 enzymes. Initially, it was thought that E2 enzymes only functioned as carriers for the activated ubiquitin by transferring it to the E3s. However, later studies found that certain E2s can also directly conjugate ubiquitin to substrates. E2 enzymes exhibit structural and functional diversity. In simple eukaryotes such as yeast (*S. cerevisiae*), there are potentially 13 genes encoding E2 enzymes, while mammals have an estimated 25–30. The diversity of E2 enzymes contributes to the specificity of the ubiquitin conjugation reaction, as E2s bind selectively to E3s. Since the number of E3 enzymes is even greater than that of E2s, the combination of different E2s and E3s allows for a high degree of substrate specificity [1].

**Figure 2:**
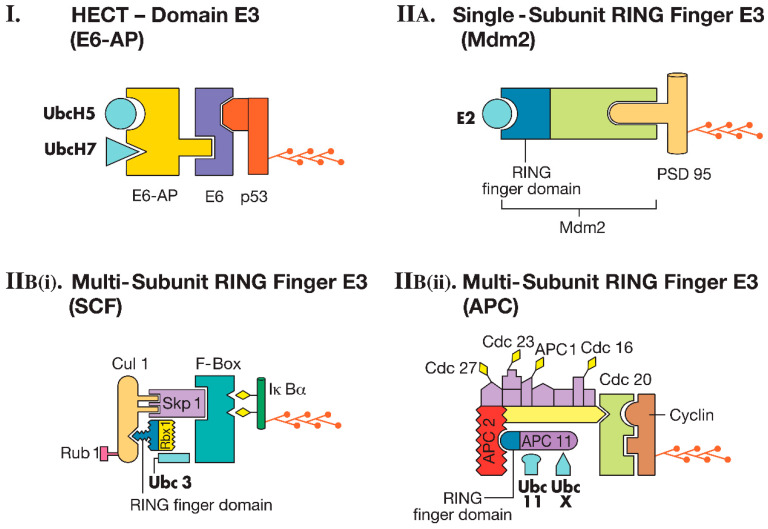
Classes of ubiquitin ligases (E3s). (**I**) HECT-Domain E3. The E6-AP ubiquitin ligase, in conjunction with the E6 protein and one of two E2 enzymes (UbcH5 or UbcH7), facilitates the attachment of ubiquitin to the p53 tumor suppressor protein. (**II**(**A**)) Single-subunit RING finger E3. Mdm2, with the assistance of an E2 enzyme, catalyzes the ubiquitination of PSD 95. (**II**(**Bi**)) Multi-subunit RING finger E3. SCF ligases feature the substrate recognition site on an F box protein. Skp1 acts as an adaptor, linking the F box protein to Cul1, and the ring finger domain is located on Rbx1. The E2 partner is Ubc3. The activity of the ligase complex is enhanced by the modification of Cul1 by Rub1, another ubiquitin-like protein. The substrate, IκBα, is phosphorylated (represented by diamonds). (**II**(**Bii**)) Multi-subunit RING finger E3. The APC complex represents a more intricate example of multi-subunit RING finger E3s and has a subunit composition distinct from that of SCF. In the APC complex, the Cdc20 protein serves as the substrate recognition site for cyclin. The RING finger domain is located on APC11. The E2 enzymes Ubc11 or UbcX can work with the APC ligase. Several adaptor proteins, some of which are labeled (Cdc27, Cdc23, APC1, Cdc16) and others unlabeled, interact with Cdc20 and APC11. Phosphorylation is indicated by diamonds on the adaptor subunits. Polyubiquitin chains are shown attached to the substrates in each panel (Figure modified from Hegde 2004 and reprinted with permission from Elsevier© 2004) [2,14,15,16].

E3s are the enzymes that specifically recognize substrates. There are about 600 ubiquitin ligases in human cells. These enzymes can be single proteins or protein complexes. Single-subunit E3s can accept ubiquitin from E2 in a thioester linkage and directly ligate it to the substrate. In contrast, multi-subunit E3s generally function by bringing the E2 and substrate together, facilitating the transfer of ubiquitin to the substrate. E3 enzymes are the most diverse among the ubiquitin-conjugating enzymes. There are two major classes of E3s: (1) HECT (homologous to E6-AP carboxyl-terminus) domain E3s and (2) RING (really interesting new gene) finger E3s [2] (Figure 2).

#### 2.2.1. HECT Domain E3s

A well-known example in the class of HECT domain E3s is the ubiquitin ligase E6-AP (later renamed Ube3a), which attaches ubiquitin to the tumor suppressor protein p53. The viral protein E6 binds to a cellular protein known as E6-AP (E6-associated protein). The catalytic domain of the ubiquitin ligase resides in the C-terminal region of E6-AP. This ligase can function with E2 enzymes UbcH5 or UbcH7. Subsequent research revealed that a family of ubiquitin ligases, homologous to the catalytic domain of E6-AP, exists. These enzymes are referred to as HECT domain E3s. Along with the HECT domain, many E3s also contain another domain called the WW domain. Furthermore, the WW domain-containing E3s frequently possess a C2 domain. The presence of the C2 domain is particularly significant in the context of the nervous system because it responds to increased intracellular calcium (Ca^2+^) levels and aids in translocation to the plasma membrane. Therefore, the inclusion of this domain in neuronal HECT E3s may play a crucial role in the ubiquitination of neurotransmitter receptors or the proteins associated with them [2,14] (Figure 2I).

#### 2.2.2. RING Finger E3s

These E3 enzymes are called RING finger E3s because they contain a RING finger domain, a structural feature composed of seven cysteine residues and one histidine residue, which together form a folded domain that binds two zinc ions. Over time, several other ubiquitin ligases with the RING finger domain have been identified. The RING finger motif in these ligases is essential for transferring ubiquitin either to substrates or to RING finger proteins themselves. RING finger E3s can be further classified into two categories: (1) single-subunit RING finger E3s and (2) multi-subunit RING finger E3s.

##### Single-Subunit RING Finger E3s

This subclass of E3s possesses both the RING finger domain and the substrate recognition site within the same protein. One of the most well-characterized single-subunit RING finger E3s is Mdm2, which ubiquitinates the protein p53 in normal cells. As previously mentioned, E6-AP, a HECT ubiquitin ligase, also ubiquitinates p53 in cells infected with human papillomavirus (HPV). It has been shown that in HPV-infected cells, E6-AP is responsible for ubiquitinating p53. Although Mdm2 is present in HPV-infected cells, it does not mediate the ubiquitination of p53 in these cells. Further studies using antisense oligonucleotides targeted at E6-AP revealed that E6-AP is essential for the degradation of p53 in HPV-positive cells but not in HPV-negative cells. Conversely, reducing the expression of Mdm2 or introducing peptides that inactivate Mdm2 led to decreased p53 degradation in HPV-negative cells, but had no effect in HPV-positive cells. The structural determinants recognized by E6-AP and Mdm2 on p53 differ from one another. For instance, in p53 targeted by E6-AP, asparagine is found at position 268, while in p53 recognized by Mdm2, aspartate is located at the same position [14] (Figure 2II(A)).

##### Multi-Subunit RING Finger E3s

*(i) SCF Complex (SKP1-Cullin-F-Box Protein Complex):* The SCF complex is composed of at least four proteins: Skp1, Cul1, Roc1/Rbx1/Hrt1, and an F-box protein. At the core of this complex is Rbx1, which contains the RING finger domain. SCF-type ligases also include a protein called cullin, which interacts with linker proteins like Skp1 to recruit substrate-interacting proteins, such as F-box proteins. There are five different cullins in mammals, and a number of F-box proteins. In budding yeast, the genome encodes 17 F-box proteins, while the human genome contains 69. Therefore, just the combination of cullins and F-box proteins alone makes it theoretically possible to create hundreds of distinct E3 ligases with varying specificities. The activity of SCF ligases is regulated by two types of post-translational modifications. The first is the covalent attachment of the ubiquitin-like protein Rub1 to cullin. The second is through the regulation of F-box protein levels by autocatalytic ubiquitin-mediated degradation. One well-studied substrate of the SCF complex is IκBα (Figure 2II(Bi)).

*(ii) Anaphase Promoting Complex (APC):* Although the APC contains a subunit with a RING finger domain (APC 11), it is distinct from the SCF ligase in terms of overall subunit composition. For instance, unlike SCF ligases, which have a single adaptor protein like Skp1, the APC includes multiple subunits that act as adaptors. Additionally, while substrate phosphorylation plays a key role in specific substrate recognition by SCF ligases, it does not appear to be a significant factor for APC substrate recognition. Instead, substrate specificity in the APC is modulated by the incorporation of ‘specificity factors’ into the ligase complex. For example, the protein Cdc20 enables the APC to degrade substrates at the onset of anaphase, such as the anaphase inhibitor Pds1p. Conversely, substituting Cdc20 with a different specificity factor, called Hct1, enables the APC to degrade a distinct set of substrates, such as mitotic cyclins, later in anaphase. The APC functions in conjunction with the E2 enzymes Ubc11 or UbcX. One of the well-characterized substrates of the APC is mitotic cyclin, which contains a short sequence of nine amino acids known as the ‘destruction box’ that is crucial for recognition by the APC ubiquitin ligase [16] (Figure 2II(Bii)).

In addition to the E3 ligase classes described above, it is possible that other types of ligases exist in nature. After the discovery of above-mentioned categories of ligases, RBR (RING-between-RING) ligases were described which also ligate ubiquitin to lysine residues [17,18]. These ligases use a combination of already described catalytic activities [18] and therefore are not truly novel.

The discovery of a new class of ligases had to await the development of new chemical probes that target the key amino acid (cysteine) in the catalytic domain of ubiquitin-conjugating enzymes. Such chemicals are called activity-based probes (ABPs). Using an ABP targeted against a protein named MYCBP2 in SH-SY neuroblastoma cell extracts, Pao et al. discovered a hitherto unknown catalytic activity for an E3 enzyme [19]. This activity requires two cysteine residues unlike the previously classified ubiquitin ligases, which require just one cysteine. In addition, MYCBP2 covalently links ubiquitin to the β hydroxyl group in the side chain of threonine instead of lysine [19]. This type of ligase has been designated RING-Cys-relay (RCR) [19] and appears to be the sole member of this class. MYCBP2 functions as a key signaling center in the nervous system, regulating synaptic growth and development as well as neuronal connectivity [20].

### 2.3. The Proteasome

**Figure 3:**
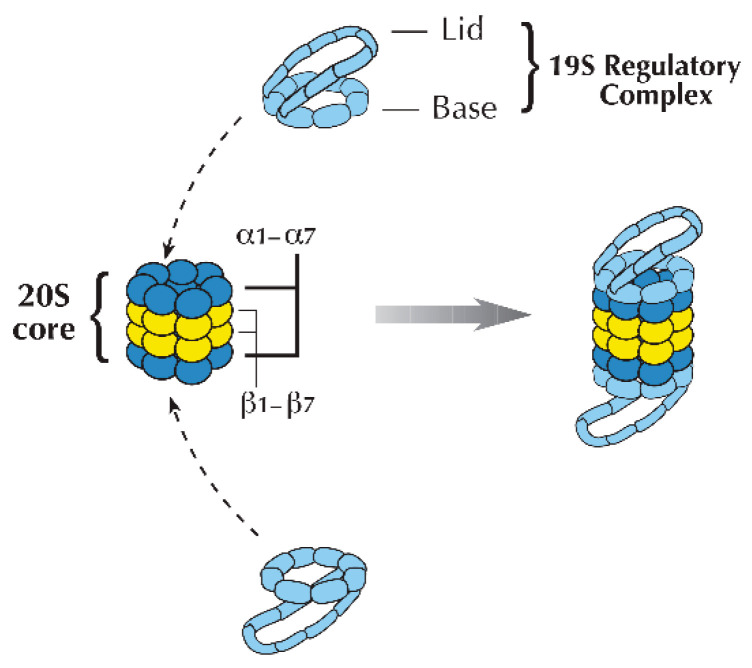
The proteasome. This proteolytic complex is composed of a 20S core particle and two 19S regulatory complexes (19S RCs), which are attached to each side of the core particle. The 20S proteasome houses the catalytic sites and consists of four stacked rings. The inner two rings are composed of seven β subunits (β1–β7), while the outer rings contain seven α subunits (α1–α7). The 19S RC is comprised of a base and a lid, each containing eight subunits (Figure modified from Hegde 2004 and reprinted with permission from Elsevier© 2004) [1,2,20].

The term “proteasome” refers to two types of multi-subunit proteolytic complexes, namely the 26S and 20S, categorized based on their sedimentation coefficient. The 26S proteasome is responsible for degrading ubiquitinated protein substrates. It consists of a cylindrical catalytic 20S core and regulatory “caps” at each end, forming a structure that resembles a dumbbell. Each cap of the 26S proteasome is known as the 19S regulatory complex (19S RC). The 19S RC functions to channel the ubiquitinated substrates into the 20S core which degrades them [20] (Figure 3).

#### 2.3.1. The Catalytic 20S Core

Our understanding of how the proteasome is put together has been derived from the studies on the crystal structures of the proteasome in the archaebacterium *Thermoplasma acidophilum* and the yeast *Saccharomyces cerevisiae*. Evidence suggests that the proteasome predates ubiquitin, as archaebacteria possess proteasomes but not ubiquitin. *T. acidophilum* has two genes that encode α and β subunits. These subunits are arranged in four stacked rings to form the catalytic cylinder, with the two middle rings composed of β subunits, positioned between two rings of α subunits. In the *T. acidophilum* proteasome, both the α and β subunits are present in seven copies each, arranged symmetrically as α7β7β7α7. This overall structure is conserved in eukaryotes, though the α and β subunits have diverged into seven different subunits for each. In yeast, the 20S core is composed of two outer rings with seven α subunits (α1 to α7) in each ring, and two inner rings made up of seven β subunits each (β1 to β7) (Figure 3).

The catalytic core of the proteasome functions as a threonine protease. The 20S proteasome can exist not only as part of the 26S complex but also as a separate entity that cannot degrade ubiquitinated proteins. Nonetheless, the 20S proteasome on its own exhibits chymotrypsin-like, trypsin-like, and postglutamyl peptidase activities, which cleave after hydrophobic, basic, and acidic residues, respectively. The peptide hydrolyzing activity of the 20S proteasome can be further modulated by an alternative regulatory cap, known as the 11S [2,20].

#### 2.3.2. The 19S Regulatory Complex (RC)

The 19S RC is responsible for recognizing polyubiquitinated substrates and guiding these substrates into the catalytic 20S core of the proteasome. Additionally, it plays a role in regulating the activity of the catalytic core and determining the nature of the degradation process. Typically, one 19S RC is attached to either end of the catalytic core. Certain subunits of the 19S RC facilitate the entry of the substrate into the catalytic chamber for degradation.

The 19S RC is composed of a base and a lid (Figure 3). The base includes six ATPase subunits (Rpt1–Rpt6) and two non-ATPase subunits (Rpn1, Rpn2, and Rpn13). The lid consists of ten non-ATPase subunits (Rpn2, Rpn5, Rpn6, Rpn7, Rpn8, Rpn9, Rpn10, Rpn11, Rpn12, and Rpn15) [20].

### 2.4. Deubiquitinating Enzymes (DUBs)

Ubiquitination is a reversible process before the ubiquitinated protein is committed to degradation by the proteasome. Ubiquitin is removed from substrates by enzymes known as deubiquitinating enzymes (DUBs). DUBs can be classified into two general categories based on their protein sequence and molecular size: (1) low-molecular-weight (20–30 kDa) ubiquitin C-terminal hydrolases (UCHs), and (2) high-molecular-weight (approximately 100 kDa) ubiquitin-specific proteases (UBPs, also called USPs). The UBP family is large, containing diverse genes, while the UCH family is smaller. For instance, in *Saccharomyces cerevisiae*, there are seventeen UBPs and one UCH. In humans, sixty-three genes encode UBPs, while four genes encode UCHs. UCHs and UBPs perform distinct roles within eukaryotic cells. While the name “deubiquitinating enzymes” emphasizes their role in removing ubiquitin from substrates, certain DUBs, especially UCHs, process linearly linked ubiquitin precursors to generate monoubiquitin. DUBs are crucial for generating free ubiquitin at various stages of the ubiquitin–proteasome pathway. Ubiquitin is encoded by a tandemly linked polyubiquitin gene. Based on the evidence available in the literature, it appears that polyubiquitin is processed by UCHs or other DUBs to produce monoubiquitin. Besides the polyubiquitin gene, ubiquitin is also encoded through fusion with two ribosomal subunits, L40 and S27. These gene products are also processed by DUBs. The cleavage of isopeptide bonds in the ubiquitin chains, linked through Lys-48, Lys-63, or other lysine residues in ubiquitin, serves two primary purposes: One function is to recycle ubiquitin after its use in the ubiquitination of a substrate. Another function is to “edit” errors made by the ubiquitin-conjugating enzymes, reversing the ubiquitination reaction so that the substrate does not undergo its targeted process, such as degradation or endocytosis [1,2].

## 3. Association Between the UPP and the Disorders of the Nervous System

The molecules of the UPP are linked to several mental disorders and neurodegenerative diseases. Of the mental disorders, we focus on Angelman Syndrome, autism, major depressive disorder, and schizophrenia. Among the neurodegenerative diseases, we have chosen to describe the UPP’s connection to amyotrophic lateral sclerosis, Huntington’s disease, Parkinson’s disease, and spinocerebellar ataxia.

### 3.1. Angelman Syndrome (AS)

Angelman Syndrome (AS) is named after Harry Angelman, an English physician who first described the condition. The symptoms of AS include intellectual disability, a notably cheerful disposition, a high susceptibility to epileptic seizures, and an abnormal gait [21]. The condition is known to affect approximately 1 in every 15,000 births. In approximately 65–75% of individuals with AS, a deletion of genetic material on the maternal chromosome 15q11-q13 is observed. Other genetic abnormalities, such as uniparental disomy and imprinting mutations, are also found in AS patients, each contributing to about 3–5% of cases. These genetic defects occur in a gene known as *UBE3A* [9,10]. Point mutations in *UBE3A* are seen in around 4–6% of AS cases [11]. *UBE3A* is a maternally imprinted gene, with expression in the brain coming exclusively from the maternal allele [22,23]. The gene encodes a ubiquitin ligase, previously identified as E6-AP ubiquitin ligase. Early studies focused on the action of E6-AP in attaching ubiquitin to p53, but it is also known to attach ubiquitin to at least three other substrates: (i) RAD23, a human counterpart of a yeast DNA repair protein [24]; (ii) multi-copy maintenance protein 7 (MCM7), which is thought to play a role in chromosome replication [25]; and (iii) E6-AP itself [26].

The intellectual disability observed in AS patients suggests a malfunction at the synaptic level. Supporting this hypothesis, mice with a deficiency of the Ube3a maternal allele show impairments in long-term potentiation (LTP) and contextual learning. Early research demonstrated an indirect effect of Ube3a on calcium/calmodulin-dependent protein kinase II (CaMKII), a molecule critical for synaptic plasticity. In the hippocampus of AS model mice, an increase in inhibitory autophosphorylation of CaMKII at Thr305 and Thr306 leads to a reduction in kinase activity and causes the dissociation of CaMKII from the postsynaptic density [27]. Additionally, reducing inhibitory autophosphorylation of CaMKII in AS mice has been shown to rescue neurological deficits in these model mice [28]. However, it remains unclear how mutations in Ube3a lead to alterations in CaMKII. Another molecule crucial for synaptic plasticity, Arc, has been identified as a substrate for Ube3a, and it has been proposed that Ube3a regulates the development of excitatory synapses by targeting Arc [29].

Recent studies showed that in AS model mice, endocytosis of small conductance potassium channel (SK2) is impaired resulting in higher number of postsynaptic SK2 molecules. This causes reduction in NMDA receptor activation and impairment of LTP. The deficit in LTP could be rescued by pharmacological inhibition of SK2 channels [30].

The other molecular target of Ube3a with a role in synaptic plasticity is p18 (aka LAMTOR1). This protein is part of a pentameric complex called the Ragulator, which maintains lysosomal localization of Rag GTPase dimers (consisting of Rag A and B). In the presence of amino acids, binding to Rag A/B dimers activates mammalian target of rapamycin complex 1 (mTORC1). Abnormal mTORC1 activation is linked to AS. Ube3a ubiquitinates p18 and targets it for degradation by the proteasome. Deficiency of Ube3a causes increases p18 amounts, which in turn causes the overactivation of mTORC1 and deficits in synaptic plasticity. Knockdown of p18 improves LTP in AS model mice [31].

Ube3a is also known to regulate the levels of Pbl/Ect2 and Ephexin5 guanine nucleotide exchanges factors for RhoA GTPases, which are critical for regulating the development of axons and dendrites [32]. Therefore, Ube3a deficiency during the development of the nervous system might have severe adverse effects on the formation of neural circuitries. This could be an explanation for some of the neurological symptoms of AS. Consistent with this idea, reactivation of the maternal allele of Ube3a in conditional AS model mice early in development rescues anxiety, repetitive behavior, and epilepsy [33].

### 3.2. Autism

The UPP is linked to autism/autism spectrum disorder (ASD) in many ways. For example, a study of postmortem brains of 13 subjects with ASD (and 13 control subjects) found that GABA_A_ α-1 protein (but not mRNA) is decreased in middle frontal gyrus of subjects with ASD. This study also observed that an endoplasmic reticulum (ER)-associated degradation (ERAD) E3 ubiquitin ligase named SYVN1 is associated with GABA_A_ α-1 protein. In addition, knocking down the expression of Syvn1 in cortical neurons of CD-1 mice increased the expression of GABA_A_ α-1 protein [34].

Another protein that links the UPP to autism is post-synaptic density protein 95 (PSD-95). One broad explanation for the mechanism underlying autism is defective activity- or experience-dependent synapse elimination during the development of the nervous system. Ubiquitination of PSD-95 is mediated by a ligase called Mdm2. A protein called protocadherin 10 (Pcdh10), which is linked to autism spectrum disorder, interacts with polyubiquitinated PSD-95 and facilitates its delivery to the proteasome for degradation [35].

A genome-wide study of 849 children with ASD and 1049 healthy ones looked at copy number variation (CNV; difference in segments of DNA) and found several new ASD susceptibility genes and genes implicated in neuronal function, such as the neuronal cell adhesion molecules *NLGN1* and *ASTN2*. This study also found that CNVs in genes of the UPP such as *UBE3A*, *PARK2*, *RFWD2*, and *FBXO40* were present in ASD cases but not in controls [36].

Regulation of the proteasome has been linked to autism through a deletion mutation in a protein called UBLPC1 (ubiquitin-like domain-containing C-terminal domain phosphatase 1). Even though this was reported from one Lebanese family, it is instructive in terms of understanding how proteolysis by the UPP might contribute to development of autism. In fibroblasts derived from affected individuals, a truncated UBLPC1 is expressed which was found to increase proteasome activity. The action of truncated UBLPC1 can be explained by understanding the function of the full-length, wild-type UBLCP1. Normally, wild-type UBLP1 dephosphorylates Serine-361 of the proteasome lid subunit Rpn1, which is required for assembly of the 26S proteasome. It also dephosphorylates multiple phosphorylation sites of an ATPase subunit in the base called Rpt1 and impairs its ATPase activity. Overall, these phosphorylation events contribute to a decrease in proteasome activity. Because truncated UBLCP1 does not have a functioning phosphatase domain owing to the deletion mutation, it is unable to dephosphorylate Rpn1 and Rpt6, which leads to increased proteasome activity. In addition, haploinsufficiency of the *PSMD12* (*RPN5*) gene, which encodes a non-ATPase subunit of the 19S RC, is found in subjects with autistic features [37].

Duplication, triplication, and gain-of-function mutation in the UBE3A gene have been linked to autism [38]. One of the missense mutations (T485A) prevents phosphorylation of Ube3a by cAMP-dependent protein kinase resulting in increased ubiquitin ligase activity [39,40]. This mutation also leads to an increase in the number of dendritic spines, which could be a contributing factor to development of the autism phenotype. Another ubiquitin ligase TRIP12 has also been linked to intellectual disability with or without ASD. Whole-exome sequencing revealed nine different alterations in the *TRIP12* gene in patients relative to controls and the changes in the gene amounted to haploinsufficiency [41].

### 3.3. Major Depressive Disorder (MDD)

MDD, also called clinical depression, is characterized by persistently depressed mood and a lack of interest in joyous activities. It might also be accompanied by disturbances in sleep, loss of appetite, and inability to think clearly.

Some studies have found an association between the components of the UPP and MDD. A gene expression study of 48 individuals with MDD found that genes encoding proteasome subunits were upregulated in the hippocampus, striatum, and Brodmann area 46 of brains of subjects with MDD. For this study, the researchers identified 37 genes based on genome-wide association studies using a large data set (135,458 cases and 344,901 controls). It appears that subunits of the 20S catalytic core, as well as those of 19RC, were among the upregulated genes [42].

Another case-control study (622 MDD patients, of whom 390 had treatment-resistant depression) conducted an association study for the proteasome subunit PSMD13 and analyzed peripheral blood samples. This study found that subjects carrying the homozygous GG genotype of PSMD13 rs3817629 had a twofold greater risk of developing treatment-resistant depression compared to those carrying the A allele of PSMD13. The same subjects also had lower levels of PSMD13 mRNA in fibroblasts [43].

A study on mice found a role for MAGE-D1(melanoma antigen gene-D1) in depression-like behaviors. MAGE-D1 associates with RING E3 ubiquitin ligases and functions to ubiquitinate the serotonin transporter protein. Deficiency of MAGE-D1, created by knockout or knockdown, causes depression-like symptoms such as decreased exploratory behavior, a decrease in social interaction, and a reduction in sucrose preference. These symptoms could be reversed by acute administration of the antidepressants sertraline and imipramine [44].

SNPs in a DUB called USP46 are associated with MDD in the Japanese population (432 MDD patients and 792 controls) [45].

### 3.4. Schizophrenia

One of the possible causes of schizophrenia is the improper arrangement of synaptic connections during development of the brain. A molecule that plays multiple roles in the nervous system called DISC1 (disrupted in schizophrenia 1) is associated with schizophrenia. DISC1 was originally identified through genetic studies of a Scottish family prone to psychiatric diseases. In this case, the abnormal DISC1 resulted from chromosomal translocation [46]. It has been suggested that the full-length DISC1 protein, when post-translationally modified, can contribute to the development of some sporadic forms of schizophrenia because of aberrant multimerization, which converts the protein into an insoluble form prone to aggregation [47].

DISC1 functions as a scaffolding protein that helps organize protein complexes and because of this, the protein has multiple functions in the nervous system. For example, DISC1 is part of a protein complex at the centrosome and plays a role in regulating the cytoskeletal processes, which in turn control neuronal migration and neurite outgrowth [48].

DISC1 itself is a substrate for ubiquitin–proteasome-mediated degradation. It is targeted for ubiquitination by an SCF-type ligase. The substrate-specific part of this SCF has been shown to be an F-Box protein called FBXW7 [49]. A key step in rendering an SCF-substrate susceptible to ubiquitination is phosphorylation. Analysis of consensus motifs for phosphorylation suggested that glycogen synthase kinase-3 (GSK3) is the putative kinase that phosphorylates DISC1. DISC1 and GSK3 appear to regulate each other. It has been demonstrated that DISC1 directly binds GSK and negatively regulates its function, leading to an increased concentration of β-catenin, which in turn boosts the proliferation of neural progenitor cells [50]. It has been shown that blocking the DISC1–FBXW7 interaction can stabilize DISC1, which can be a potential therapeutic strategy to boost the levels of DISC1 in cases of DISC1 haploinsufficiency where one copy of the gene is absent because of some genetic abnormality [49].

A protein that interacts with DISC1 is FEZ1 (fasciculation and elongation protein Zeta-1). Many studies have shown that FEZ1 plays a role in neuronal development and the formation of synapses in the central nervous system as well as the peripheral nervous system. In the mouse hippocampus, Fez1 synergistically functions with Disc1 to regulate dendritic development in newborn neurons in the dentate gyrus. Fez1 is targeted for ubiquitination by the anaphase-promoting complex (Cdc20) ligase [51].

Defective development of neural circuits as relevant to schizophrenia could also be potentially mediated by other molecules linked to the UPP. A study on induced pluripotent stem cells derived from patients with exon 8 mutations and a 4 bp deletion in exon 12 showed that impaired neurite outgrowth is attributable to a decrease in a netrin receptor called UNC5D [52]. Although so far there has not been any direct connection to the UPP, another netrin receptor UNC-40 was shown to be a substrate for ubiquitin–proteasome-mediated degradation [53].

Several studies on postmortem brains of schizophrenia patients and matched controls investigated the expression of components of the UPP. Some studies have found a decrease in mRNAs of ubiquitin ligases such as UBE3B [54] and FBXW7 [55], while other studies of ubiquitin ligase proteins found decreases in the amounts of UBE3B, FBXL21, and MDM2 [54,56] in the dorsolateral prefrontal cortex of subjects with schizophrenia. A recent exome-sequencing study of four families with schizophrenia found five missense mutations in the *TULP4* gene which encodes a novel ubiquitin ligase. Knockdown of Tulp4 in mice causes delayed neuronal migration in the cerebral cortex and leads to impairment of sensory-motor gating and spatial learning in adult mice [57]. Among the DUBs, UCHL1 UCHL5, USP14, and USP9 were found to be decreased in the prefrontal cortex of subjects with schizophrenia [56,58,59,60].

Other investigations focused on a possible association between proteasome subunits and schizophrenia. A study on postmortem brain samples of elderly subjects with schizophrenia found decreased expression of three subunits of the 19S regulatory complex of the proteasome, namely Rpt1, Rpt2, and Rpt6. This study also found decreased levels of the α subunit of an alternative 11S regulatory complex that regulates the degradation of small peptides [61].

Another study carried out transcriptome analysis on the superior temporal gyrus and found a downregulation of 12 proteasome subunits. These data were replicated in six cohorts overall with 267 schizophrenia and 266 control samples. It is interesting to note that half of the downregulated genes encode subunits of the catalytic core of the proteasome. The other half is equally split between the ATPase and non-ATPase subunits of the regulatory cap of the proteasome. Therefore, it appears that in a subtype of schizophrenia, the channeling of polyubiquitinated substrates into the catalytic core *and* the degradation of channeled substrates are both impaired [62].

Levels of ubiquitinated proteins appear to be altered in the brains of subjects with schizophrenia as well. An investigation of the postmortem orbitofrontal cortex of 38 schizophrenia patients and 38 matched controls found increased levels of ubiquitinated proteins. The same study also found that erythrocytes from patients with treatment-resistant schizophrenia also had increased levels of ubiquitinated proteins [63]. In contrast, a previous study on the postmortem superior temporal gyrus found decreased levels of K-48-linked polyubiquitination [64], which is the one that marks substrate proteins for proteasome-mediated degradation. A simple explanation for these disparate findings might be that the two studies tested different brain regions, although a more likely explanation is a technical one. Bousman et al.’s (2019) study [63] did not test for different types of polyubiquitin conjugates such as K48- and K63-linked polyubiquitination. Rubio et al.’s study [64] does not provide enough methodological details. Therefore, it is not clear whether their experiments indeed were discriminating between K48- and K63-linked polyubiquitination.

It is possible that dysregulation of proteasome function varies between intracellular compartments. A study on postmortem superior temporal gyrus of 25 schizophrenia and 25 control subjects measured proteasome activity using fluorescent substrates. The researchers found that trypsin-like activity was decreased in the nucleus and chymotrypsin-like activity was lower in the cytosolic fraction in the schizophrenia samples relative to controls [65]. A caveat for this study is that the catalytic activities assessed reflect the activity of the 20S catalytic core and not degradation of the polyubiquitinated substrates by the 26S proteasome, which is a more meaningful measure with respect to degradation of cellular proteins.

Some genetic studies have shown an association between single-nucleotide polymorphisms between schizophrenia and some genes that encode components of the UPP. In Irish families with high incidence of schizophrenia (814 cases and 625 controls), single-nucleotide polymorphisms (SNPs) in the *FBXL21* gene at two loci (rs1859427 and rs6861170) were found [66]. The *FBXL21* gene encodes an F-Box protein, which is the substrate-binding protein in an SCF ligase. The FBXL2 protein targets NMDA receptors for ubiquitination and degradation [67]. A case-control study of the Chinese Han population (296 cases, 320 controls) showed an association of the pathogenesis of schizophrenia with SNPs of the *NEDD4* gene at two loci (rs3088077 and rs2303579), while cognitive dysfunctions were associated with SNPs in the *NEDD4* gene at two other loci (rs2303579 and rs62043855) [68]. The NEDD4 protein targets the AMPA receptor for ubiquitination [69]. Another investigation (392 cases, 572 controls) observed that combinations of SNPs in the RNF4 (RING finger protein 4) gene (rs1203860 and rs2282765) and in the SART3 (squamous cell carcinoma antigen recognized by T cells 3) gene (rs2287550) were associated with an increased risk of schizophrenia [70]. The RNF4 and SART3 proteins have a role in the growth and development of the nervous system and alterations in them might contribute to development of schizophrenia through the defective development of cortical circuits [70].

### 3.5. Amyotrophic Lateral Sclerosis (ALS)

ALS affects motor neurons, which leads to a decrease in mobility among people with this condition. As the disease progresses, motor neurons degenerate, causing muscle atrophy, further exacerbating the impaired movement of patients. Mutations in *SOD1* (*Copper-zinc superoxide dismutase 1*) [71], *TDP-43* (*the gene encoding transactive response DNA binding-protein 43 kDa*) [72,73] and FUS (fused in sarcoma) [74,75] have been linked to ALS [76,77,78,79,80,81,82,83,84,85,86,87,88].

Mutations in *SOD1* are linked to certain instances of autosomal dominant familial ALS as well as some cases of sporadic ALS. Numerous studies have suggested a connection between the ubiquitin–proteasome pathway (UPP) and the turnover of SOD1, though the findings have not provided a definitive explanation. Some research has reported that mutant SOD1 proteins are degraded more quickly than wild-type SOD1 by the UPP [78,79]. In support of this, the ubiquitin ligases dorfin and NEDL1 have been shown to ubiquitinate mutant SOD1, but not wild-type SOD1. On the other hand, another study found that metal-free SOD1 is degraded by the 20S proteasome in vitro without the need for ubiquitination. This study showed that both wild-type and mutant SOD1 in their monomeric forms were susceptible to degradation by the proteasome [80]. Other reports have indicated that both wild-type and mutant SOD1 proteins undergo similar degradation through macroautophagy and the proteasome [81].

Overexpression of dorfin, a putative E3 ligase for SOD1, has been shown to reduce cell death induced by the mutant SOD1 protein [78]. Gene expression profiling of spinal cord tissue from patients with sporadic ALS suggested that genes that encode the components of the UPP, such as dorfin and ubiquitin-like protein 5, as well as genes related to oxidative damage, transcription, neuronal differentiation, and inflammation, may play a role in the pathogenesis of sporadic ALS [82]. It is possible that the expression of dorfin is increased because of a cellular response to improve the clearance of mutant SOD1. Additionally, other studies have indicated that heat-shock proteins like Hsp70 or Hsc70, along with CHIP, contribute to the proteasomal degradation of mutant SOD1 [83]. Furthermore, research has shown that oxidative damage enhances the ubiquitination of mutant SOD1 and decreases proteasome activity after one week of expression of a mutant *SOD1* gene in cultured cells [83].

The toxicity of mutant SOD1 protein aggregates remains a subject of debate. Some researchers have found that aggregates of mutant SOD1 do not lead to cell death [84], and other studies observed no difference in the viability of motor neurons from wild-type animals and those from transgenic mice expressing mutant SOD1 [85]. However, some studies have demonstrated that inhibiting the proteasome results in increased cell death in human cells expressing mutant SOD1 [86,87]. One study also suggested that SOD1 mutations may impair the proteasome function. In transgenic mice with the G93A mutation in the *SOD1* gene, the expression of proteasome subunits is reduced, leading to impaired UPP function in spinal motor neurons [88]. Despite these findings, the role of protein aggregates in familial ALS remains unclear. Like other neurodegenerative diseases, the precise relationship between SOD1 protein aggregates, the UPP, and the progression of the disease is still to be fully understood.

SOD1 is also a target for ubiquitination for other E3 ligases. In studies using HEK293 cells, researchers showed that mutant SOD1 is a substrate for an endoplasmic reticulum-associated E3 ubiquitin ligase called glycoprotein 78 (Gp78). Ubiquitination by Gp78 targets mutant SOD1 for degradation by the proteasome, which represses aggregate formation and protects the cells from cell death induced by mutant SOD1 [89]. Another E3 ligase called Smurf1 (Smad ubiquitination regulatory factor 1) was shown to promote K63-type ubiquitin attachment to SOD1, which enhanced aggresome formation and eventual autophagic degradation of misfolded SOD1 in a mouse neuroblastoma cell line (Neuro-2a, aka N2a) and a human neuroblastoma cell line SH-SY5Y [90]. A mitochondrial ligase (named MITOL) localized to the outer membrane of mitochondria ubiquitinates mutant SOD1 but not wild-type SOD1 in Neuro-2a cells. This action of MITOL was shown to reduce accumulation of mutant SOD1 in mitochondria and suppress generation of reactive oxygen species [91].

TDP-43 (transactive response DNA-binding protein 43 kDa) was initially identified in the ubiquitinated aggregates in the postmortem hippocampus, neocortex, and spinal cord tissues obtained from subjects with ALS. A few E3 ubiquitin ligases such as parkin [92], Rnf220 [93], Znf179 [94], and Praja1 [95], and an E2 called UBE2E [96], have been shown to ubiquitinate TDP-43. Evidence for possible causative roles for deficient ubiquitination in ALS has been recently obtained. Mice with haploinsufficiency of ubiquitin ligase RNf220 exhibited ALS-like symptoms such as decreased mobility. These mice also showed accumulation of TDP-43 in spinal motor neurons, muscle denervation, and atrophy, which are hallmarks of ALS pathology [97]. Therefore, it is possible that impairment of the UPP might play a role in neurodegeneration caused by TDP-43 dysfunction, as has been suggested previously [98,99].

Links between FUS and UPP in the context of ALS have also been investigated. When mutant forms of FUS commonly seen in ALS are expressed in a mouse motor neuron-like hybrid cell line (NSC-34), dysfunction of UPP was observed, as evidenced by accumulation of ubiquitinated FUS aggregates and reduction in free ubiquitin pool [100].

### 3.6. Huntington’s Disease (HD)

Huntington’s disease (HD) is a condition caused by mutations in the huntingtin gene [101]. Specifically, it arises from an abnormal expansion of CAG repeats, which code for long sequences of glutamine (polyglutamine or polyQ) [101,102,103].

Early research into HD suggested that the UPP may be compromised in this disease. For instance, when huntingtin with a 103-glutamine stretch was expressed in human embryonic kidney (HEK) 293 cells, it led to the formation of aggregates, the accumulation of ubiquitinated proteins, and cell cycle arrest. In contrast, expressing a shorter polyglutamine stretch (25 glutamines) resulted in significantly less impact on these outcomes [104]. Further evidence of UPP dysfunction came from a study utilizing mass spectrometry techniques to specifically identify polyubiquitin chains formed through the Lys-48 linkage of ubiquitin. This study revealed an accumulation of polyubiquitin chains in HD. Additionally, it was found that Lys-48 linked ubiquitin chains accumulated in the brains of R6/2 transgenic mice (a commonly used HD mouse model), a knock-in mouse model (Q150/Q150) of HD, and in the brains of individuals with HD [103].

Recent research showed that in HD, several enzymes of the UPP are associated with mutant huntingtin (mHTT). We particularly focus on a few ubiquitin ligases here because this class of enzymes targets substrates specifically for ubiquitination and subsequent degradation by the proteasome. A HECT domain ligase called Ube3a targets mHTT for K-48-linked ubiquitination, as shown by experiments using HEK293 cells [104]. In addition, Ube3a was also found to be associated with mHTT in the striatum of a knock-in mouse model of HD [104]. Ube3a knockdown exacerbated the HD phenotype in mice and reduced their life span [105]. Other studies on cell lines stably expressing mHTT with 150-polyglutamine showed that Ube3a can target these proteins for proteasome-mediated degradation [106].

Another HECT domain ligase called UBR5 has been linked to age of onset of HD in genome-wide association studies [107]. Since HTT is degraded by the proteasome, UBR5 is likely to mark HTT for degradation through Lys-48 ubiquitin linkages. In induced pluripotent stem cells (iPSCs) from HD patients, UBR5 is expressed at high levels and knockdown of its expression increases the levels of HTT [108]. Experiments on a *C. elegans* model of polyQ expansion (containing 35 polyQ repeats) showed that knockdown of Ubr5 increased polyQ aggregation and neurotoxicity [108].

An ER-associated ubiquitin ligase called HRD1 interacts with wild-type HTT as well as mutant HTT (mHTT) and ubiquitinates them [109]. The ubiquitinated HTTs are then pulled out of the ER by VCP/p97 and delivered to the proteasome for degradation. HRD1 preferentially ubiquitinates mHTTs with expanded polyQ and the overexpression of the ligase reduces mHTT aggregates [109].

Overall, it appears that mHtTT can be a substrate for the UPP and thus proteolysis can potentially keep the mutant protein in check. Given that the disease progression still occurs in the presence of ubiquitin ligases, it is possible that the UPP is not able meet the increased need for proteolysis. Therefore, improving ubiquitination of mHTT and its degradation by the proteasome could be a potential therapeutic strategy.

### 3.7. Parkinson’s Disease (PD)

The majority of the PD cases are sporadic in nature. About 15% of the cases are familial. The UPP is linked to both sporadic and familial types of PD. Because the familial type of PD has a clearly identifiable genetic connection, the role of UPP has been extensively studied in this type of heritable disease. Mutations in *SNCA* [110], *PARK2* [111], *PARK7* [112], *LRRK2* [113], *PINK1* [114], and *ATP13A2* [115] genes are linked to PD. The proteins encoded by all these genes are associated with the UPP in one way or another.

*SNCA* encodes a protein called α-synuclein, which is part of the Lewy bodies, the intracellular inclusions seen in the brains of Parkinson’s disease patients. Lewy bodies contain high amounts of ubiquitinated proteins, including ubiquitinated α-synuclein [116]. *PARK2* encodes parkin, which is an RBR-type ubiquitin ligase. The parkin protein also has a ubiquitin-like domain at its N-terminus [117].

PARK 7 is also known as DJ-1, the protein product of which is a target for SUMO-1 (small ubiquitin-like modifier 1). Conjugation to SUMO-1 is essential for the neuroprotective activity of DJ-1 [118].

Dominant mutations of *leucine-rich repeat kinase-2* (*LRRK2*) are the most predominant causes of inherited form of late-onset PD. LRRK2 protein is targeted for ubiquitination by the E3 ligase carboxyl terminus of HSP70-interacting protein (CHIP) [119,120].

Mutations in *PINK1* (*PTEN-induced putative kinase 1*) are the second most common (after those of *Parkin*) cause of autosomal recessive early-onset type of PD. Studies have shown that PINK1 protein is functionally linked to the action of parkin. The element that links the two causes of PD is a transcription repressor Zinc-finger protein 746 (Znf746), which is also known as a parkin-interacting substrate (PARIS) [121]. PINK1 phosphorylates two serine residues (322 and 613) in Znf746 which facilitates its ubiquitination by parkin. Many mutations in PINK1 abolish its kinase activity. Therefore, it is likely that PINK1 mutations lead to an accumulation of Znf746. Given that Znf746 represses the promoter of proliferator-activated receptor gamma coactivator-1-alpha (PGC-1α), a protein critical for survival of dopaminergic neurons, prevention of its degradation thus causes loss of dopaminergic neurons in substantia nigra. This has been borne out by experiments showing that conditional knockout of PINK1 in adult mice leads to progressive loss of dopaminergic neurons in a Znf746-dependent manner [121].

ATP13A2 is expressed in lysosomes and functions as a polyamine and metal ion transporter. One of its roles is reduction of abnormal α-synuclein. In experiments on SH-SY5Y cells, reduction of α-synuclein multimerization was dependent on the activity of the UPP [122].

Another gene implicated in both familial and sporadic PD is *UCH-L1* [123], which encodes a deubiquitinating enzyme called ubiquitin C-terminal hydrolase. UCH-L1 protein co-aggregates with α-synuclein in Lewy bodies, where it is modified by S-nitrosylation, leading to its destabilization and consequent α-synuclein aggregation [124]. A mutation I193M is linked to familial PD and the mutated UCH-L1 has reduced enzymatic activity. In mouse models, this mutated form causes PD-like symptoms and degeneration of dopaminergic neurons [125].

A key feature of PD is neurodegeneration. Among the proteins linked to PD, parkin has been shown to be a key regulator of the aggresome–autophagy pathway [126,127,128]. Parkin targets several misfolded proteins to the aggresome–autophagy pathway through Lys-63 linked polyubiquitination. In addition, parkin and Pink1 regulate mitophagy, which is the removal of mitochondria, particularly damaged ones. Parkin aids in mitophagy by ubiquitinating several mitochondrial outer membrane proteins that play a role in mitochondrial fusion and dynamics such as Mitofusin 1 (Mfn1), Mitofusin 2 (Mfn2), Miro1, Miro2, and Tom20v [129]. Parkin marks these proteins by attaching polyubiquitin chains with linkages through Lys-48, Lys-63, Lys-6, and Lys-11 residues in the ubiquitin molecule [130,131,132,133,134,135,136,137,138].

Parkin plays a role in promoting the ubiquitination and degradation of polyglutamine-expanded ataxin-3, thereby reducing its cellular toxicity [131,132]. Ataxin-3, which is associated with Machado–Joseph disease (MJD) or spinocerebellar ataxia-3 (SCA3), functions as a deubiquitinating enzyme (DUB) that primarily cleaves Lys-63 linkages. The DUB activity of ataxin-3 is enhanced by its own ubiquitination [134]. Both parkin and ataxin-3 are linked to the formation of aggresomes, structures that help remove misfolded proteins via autophagy. It would be of interest to explore the potential role of ubiquitination and deubiquitination activities of these proteins in regulating their own turnover, as well as their role in the early synaptic failure observed in PD or MJD/SCA3. Such investigations could also offer insights into the parkinsonian symptoms that are observed in MJD patients [136].

A connection between proteasome dysfunction and neurodegeneration in PD was demonstrated using a sophisticated mouse model, which involved the targeted conditional depletion of the 26S proteasome by inactivating a 19S proteasome subunit, Psmc1 (also known as Rpt2/S4). In this model, the depletion of the 26S proteasome resulted in neurodegeneration and the formation of Lewy body-like inclusions. These inclusions, found in the brain of the mice, contained both ubiquitin and α-synuclein, and their appearance closely resembled Lewy bodies found in the brains of human PD patients [137]. Further evidence linking the proteasome to PD was provided by a genetic study of German PD patients, which revealed that variations in intron 5 of the gene encoding the proteasome subunit S6 ATPase (now called Rpt3) were more frequently found in early-onset PD patients compared to those with late-onset PD [138]. Support for the role of another ATPase subunit, Rpt2, in PD came from the *Drosophila* model, in which Rpt2 knockdown in the CNS causes a decrease in proteasomal activity, increases the amount of insoluble ubiquitinated protein, and induces motor and non-motor phenotypes, which are believed to be Parkinson’s disease-like symptoms in the fly model [139].

### 3.8. Spinocerebellar Ataxia (SCA)

There are several types of SCA, but the common types are SCA1, SCA2, SCA3, and SCA6, and we focus on SCA3 because of its connection to the UPP. SCA3 is also known as Machado–Joseph disease and is inherited in an autosomal dominant manner. SCA3 is caused by a CAG expansion in a gene called *ATAXIN3* [140]. The normal *ATAXIN3* contains 13 to 36 CAG repeats whereas the mutant contains 68 to 79 repeats. Thus, the mutant ATAXIN3 protein contains an expanded poly glutamine (polyQ) tract. The normal ATAXIN3 protein functions as a deubiquitinating enzyme. Consistent with this idea, knockout of the *Ataxin3* gene in mice leads to accumulation of polyubiquitinated proteins [141].

Several substrates for the deubiquitinating activity of Ataxin3 are known, including the E3 ligase parkin, but a connection between these substrates and SCA has not been convincingly established. The substate of Ataxin3 highly relevant to SCA is valosin-containing proteins or ATPase p97 belonging to the super family of ATPases associated with diverse cellular activities (VCP/p97) [142]. The VCP/p97 protein functions in proteasome-mediated degradation as well as endoplasmic reticulum-associated degradation (ERAD). In the case of ERAD, the function of VCP/p97 appears to be in aiding the removal of misfolded proteins from the ER and their subsequent degradation. The mutant Ataxin3 with polyQ expansion causes accumulation of ERAD substrates. In the *Drosophila* model, the interaction of mutant Ataxin3 with VCP/p97 exacerbates its aggregation and toxicity, and disrupting this interaction ameliorates SCA phenotype in the eyes of flies [143].

An additional DUB, known as ubiquitin-specific protease 14, has also been associated with ataxia. Mice with homozygous recessive mutations in Usp14 begin to exhibit ataxia and pronounced tremors by 2–3 weeks of age. This is followed by hindlimb paralysis and death, typically occurring between 6 and 10 weeks of age [144]. These mutations drastically reduce Usp14 expression to approximately 5–10% of the levels observed in wild-type mice. The primary function of Usp14 is thought to be the recycling of ubiquitin by breaking down polyubiquitin chains into their monomeric form. Supporting this, the brains of Usp14 mutant mice show reduced levels of monomeric ubiquitin [145]. However, unlike other neurodegenerative diseases, such as Parkinson’s disease and SCA1 in humans or gad in mice, these mutant mice do not display ubiquitin-positive protein aggregates or neuronal cell loss in the CNS. Instead, these mice experience disruptions in synaptic transmission both in the central and peripheral nervous systems. The growth defects and lethality associated with the mutation can be rescued by neuron-specific expression of Usp14 [146]. While this transgene expression also partially rescues motor defects, some issues with motor coordination persist. The incomplete rescue of motor defects has been attributed to the absence of Usp14 transgene expression in cerebellar Purkinje cells [146]. Further research has shown that Usp14 plays a vital role in maintaining synaptic ubiquitin levels at neuromuscular junctions [147].

## 4. Conclusions and Future Directions

A key challenge for future research is to elucidate the precise mechanistic connection between alterations in the UPP and development of a specific CNS disorder. In some cases, the path to a mechanistic understanding seems straightforward. For example, proteolysis of DISC1 during development of the nervous system can contribute to its insufficiency and result in the aberrant formation of neural circuitry, which is a contributory factor to the development of schizophrenia. Similarly, deficient ubiquitination and proteolysis of the serotonin transporter protein, caused by low levels of MAGE-D, perhaps contributes to depression-like symptoms in mice through the excessive removal of serotonin from the synaptic cleft.

One way to advance our understanding of the pathological processes of CNS disorders is through the development of animal models, because such models allow researchers to establish cause-and-effect relationships. Ideally, studies carried out with animal models should be complemented with research on postmortem brain samples and studies on induced pluripotent stems cells obtained from patient populations. A thorough understanding of the role of the UPP in specific mental disorders and neurodegenerative diseases can lead to the discovery of small molecules for therapeutic purposes.

## References

[1] Glickman M.H., Ciechanover A. (2002). The ubiquitin-proteasome proteolytic pathway: Destruction for the sake of construction. Physiol. Rev..

[2] Hegde A.N. (2004). Ubiquitin-proteasome-mediated local protein degradation and synaptic plasticity. Prog. Neurobiol..

[3] Spano D., Catara G. (2023). Targeting the Ubiquitin-Proteasome System and Recent Advances in Cancer Therapy. Cells.

[4] Hegde A.N., Upadhya S.C. (2007). The ubiquitin-proteasome pathway in health and disease of the nervous system. Trends Neurosci..

[5] Cole G.M., Timiras P.S. (1987). Ubiquitin-protein conjugates in Alzheimer’s lesions. Neurosci. Lett..

[6] Glotzer M., Murray A.W., Kirschner M.W. (1991). Cyclin is degraded by the ubiquitin pathway. Nature.

[7] Shabbeer S., Omer D., Berneman D., Weitzman O., Alpaugh A., Pietraszkiewicz A., Metsuyanim S., Shainskaya A., Papa M.Z., Yarden R.I. (2013). BRCA1 targets G2/M cell cycle proteins for ubiquitination and proteasomal degradation. Oncogene.

[8] Paccosi E., Artemi G., Filippi S., Balzerano A., Costanzo F., Laghezza-Masci V., Proietti S., Proietti-De-Santis L. (2023). Cockayne syndrome group A protein localizes at centrosomes during mitosis and regulates Cyclin B1 ubiquitination. Eur. J. Cell Biol..

[9] Kishino T., Lalande M., Wagstaff J. (1997). UBE3A/E6-AP mutations cause Angelman syndrome. Nat. Genet..

[10] Matsuura T., Sutcliffe J.S., Fang P., Galjaard R.J., Jiang Y.H., Benton C.S., Rommens J.M., Beaudet A.L. (1997). De novo truncating mutations in E6-AP ubiquitin-protein ligase gene (UBE3A) in Angelman syndrome. Nat. Genet..

[11] Jiang Y.H., Armstrong D., Albrecht U., Atkins C.M., Noebels J.L., Eichele G., Sweatt J.D., Beaudet A.L. (1998). Mutation of the Angelman ubiquitin ligase in mice causes increased cytoplasmic p53 and deficits of contextual learning and long-term potentiation. Neuron.

[12] Hegde A.N. (2010). The ubiquitin-proteasome pathway and synaptic plasticity. Learn. Mem..

[13] Fu H., Reis N., Lee Y., Glickman M.H., Vierstra R.D. (2001). Subunit interaction maps for the regulatory particle of the 26S proteasome and the COP9 signalosome. EMBO J..

[14] Hengstermann A., Linares L.K., Ciechanover A., Whitaker N.J., Scheffner M. (2001). Complete switch from Mdm2 to human papillomavirus E6-mediated degradation of p53 in cervical cancer cells. Proc. Natl. Acad. Sci. USA.

[15] Deshaies R.J. (1999). SCF and Cullin/Ring H2-based ubiquitin ligases. Annu. Rev. Cell Dev. Biol..

[16] Page A.M., Hieter P. (1999). The anaphase-promoting complex: New subunits and regulators. Annu. Rev. Biochem..

[17] Wang X.S., Cotton T.R., Trevelyan S.J., Richardson L.W., Lee W.T., Silke J., Lechtenberg B.C. (2023). The unifying catalytic mechanism of the RING-between-RING E3 ubiquitin ligase family. Nat. Commun..

[18] Riley B.E., Lougheed J.C., Callaway K., Velasquez M., Brecht E., Nguyen L., Shaler T., Walker D., Yang Y., Regnstrom K. (2013). Structure and function of Parkin E3 ubiquitin ligase reveals aspects of RING and HECT ligases. Nat. Commun..

[19] Pao K.C., Wood N.T., Knebel A., Rafie K., Stanley M., Mabbitt P.D., Sundaramoorthy R., Hofmann K., van Aalten D.M.F., Virdee S. (2018). Activity-based E3 ligase profiling uncovers an E3 ligase with esterification activity. Nature.

[20] Tanaka K. (2009). The proteasome: Overview of structure and functions. Proc. Jpn. Acad. Ser. B Phys. Biol. Sci..

[21] Williams C.A. (2005). Neurological aspects of the Angelman syndrome. Brain Dev..

[22] Albrecht U., Sutcliffe J.S., Cattanach B.M., Beechey C.V., Armstrong D., Eichele G., Beaudet A.L. (1997). Imprinted expression of the murine Angelman syndrome gene, Ube3a, in hippocampal and Purkinje neurons. Nat. Genet..

[23] Rougeulle C., Glatt H., Lalande M. (1997). The Angelman syndrome candidate gene, UBE3A/E6-AP, is imprinted in brain. Nat. Genet..

[24] Kumar S., Talis A.L., Howley P.M. (1999). Identification of HHR23A as a substrate for E6-associated protein-mediated ubiquitination. J. Biol. Chem..

[25] Kühne C., Banks L. (1998). E3-ubiquitin ligase/E6-AP links multicopy maintenance protein 7 to the ubiquitination pathway by a novel motif, the L2G box. J. Biol. Chem..

[26] Nuber U., Schwarz S.E., Scheffner M. (1998). The ubiquitin-protein ligase E6-associated protein (E6-AP) serves as its own substrate. Eur. J. Biochem..

[27] Weeber E.J., Jiang Y.H., Elgersma Y., Varga A.W., Carrasquillo Y., Brown S.E., Christian J.M., Mirnikjoo B., Silva A., Beaudet A.L. (2003). Derangements of hippocampal calcium/calmodulin-dependent protein kinase II in a mouse model for Angelman mental retardation syndrome. J. Neurosci..

[28] van Woerden G.M., Harris K.D., Hojjati M.R., Gustin R.M., Qiu S., de Avila Freire R., Jiang Y.H., Elgersma Y., Weeber E.J. (2007). Rescue of neurological deficits in a mouse model for Angelman syndrome by reduction of alphaCaMKII inhibitory phosphorylation. Nat. Neurosci..

[29] Greer P.L., Hanayama R., Bloodgood B.L., Mardinly A.R., Lipton D.M., Flavell S.W., Kim T.K., Griffith E.C., Waldon Z., Maehr R. (2010). The Angelman Syndrome protein Ube3A regulates synapse development by ubiquitinating arc. Cell.

[30] Sun J., Zhu G., Liu Y., Standley S., Ji A., Tunuguntla R., Wang Y., Claus C., Luo Y., Baudry M. (2015). UBE3A Regulates Synaptic Plasticity and Learning and Memory by Controlling SK2 Channel Endocytosis. Cell Rep..

[31] Sun J., Liu Y., Jia Y., Hao X., Lin W.J., Tran J., Lynch G., Baudry M., Bi X. (2018). UBE3A-mediated p18/LAMTOR1 ubiquitination and degradation regulate mTORC1 activity and synaptic plasticity. eLife.

[32] Reiter L.T., Seagroves T.N., Bowers M., Bier E. (2006). Expression of the Rho-GEF Pbl/ECT2 is regulated by the UBE3A E3 ubiquitin ligase. Hum. Mol. Genet..

[33] Silva-Santos S., van Woerden G.M., Bruinsma C.F., Mientjes E., Jolfaei M.A., Distel B., Kushner S.A., Elgersma Y. (2015). Ube3a reinstatement identifies distinct developmental windows in a murine Angelman syndrome model. J. Clin. Investig..

[34] Crider A., Pandya C.D., Peter D., Ahmed A.O., Pillai A. (2014). Ubiquitin-proteasome dependent degradation of GABAAα1 in autism spectrum disorder. Mol. Autism.

[35] Tsai N.P., Wilkerson J.R., Guo W., Maksimova M.A., DeMartino G.N., Cowan C.W., Huber K.M. (2012). Multiple autism-linked genes mediate synapse elimination via proteasomal degradation of a synaptic scaffold PSD-95. Cell.

[36] Glessner J.T., Wang K., Cai G., Korvatska O., Kim C.E., Wood S., Zhang H., Estes A., Brune C.W., Bradfield J.P. (2009). Autism genome-wide copy number variation reveals ubiquitin and neuronal genes. Nature.

[37] Soueid J., Hamze Z., Bedran J., Chahrour M., Boustany R.M. (2023). A novel autism-associated UBLCP1 mutation impacts proteasome regulation/activity. Transl. Psychiatry.

[38] Copping N.A., Christian S.G.B., Ritter D.J., Islam M.S., Buscher N., Zolkowska D., Pride M.C., Berg E.L., LaSalle J.M., Ellegood J. (2017). Neuronal overexpression of Ube3a isoform 2 causes behavioral impairments and neuroanatomical pathology relevant to 15q11.2-q13.3 duplication syndrome. Hum. Mol. Genet..

[39] Yi J.J., Berrios J., Newbern J.M., Snider W.D., Philpot B.D., Hahn K.M., Zylka M.J. (2015). An Autism-Linked Mutation Disables Phosphorylation Control of UBE3A. Cell.

[40] Xing L., Simon J.M., Ptacek T.S., Yi J.J., Loo L., Mao H., Wolter J.M., McCoy E.S., Paranjape S.R., Taylor-Blake B. (2023). Autism-linked UBE3A gain-of-function mutation causes interneuron and behavioral phenotypes when inherited maternally or paternally in mice. Cell Rep..

[41] Zhang J., Gambin T., Yuan B., Szafranski P., Rosenfeld J.A., Balwi M.A., Alswaid A., Al-Gazali L., Shamsi A.M.A., Komara M. (2017). Haploinsufficiency of the E3 ubiquitin-protein ligase gene TRIP12 causes intellectual disability with or without autism spectrum disorders, speech delay, and dysmorphic features. Hum. Genet..

[42] Belaish S., Israel-Elgali I., Shapira G., Krieger I., Segev A., Nitzan U., Majer M., Bloch Y., Weizman A., Gurwitz D. (2021). Genome wide analysis implicates upregulation of proteasome pathway in major depressive disorder. Transl. Psychiatry.

[43] Minelli A., Magri C., Barbon A., Bonvicini C., Segala M., Congiu C., Bignotti S., Milanesi E., Trabucchi L., Cattane N. (2015). Proteasome system dysregulation and treatment resistance mechanisms in major depressive disorder. Transl. Psychiatry.

[44] Mouri A., Sasaki A., Watanabe K., Sogawa C., Kitayama S., Mamiya T., Miyamoto Y., Yamada K., Noda Y., Nabeshima T. (2012). MAGE-D1 regulates expression of depression-like behavior through serotonin transporter ubiquitylation. J. Neurosci..

[45] Fukuo Y., Kishi T., Kushima I., Yoshimura R., Okochi T., Kitajima T., Matsunaga S., Kawashima K., Umene-Nakano W., Naitoh H. (2011). Possible association between ubiquitin-specific peptidase 46 gene and major depressive disorders in the Japanese population. J. Affect. Disord..

[46] St Clair D., Blackwood D., Muir W., Carothers A., Walker M., Spowart G., Gosden C., Evans H.J. (1990). Association within a family of a balanced autosomal translocation with major mental illness. Lancet.

[47] Leliveld S.R., Hendriks P., Michel M., Sajnani G., Bader V., Trossbach S., Prikulis I., Hartmann R., Jonas E., Willbold D. (2009). Oligomer assembly of the C-terminal DISC1 domain (640–854) is controlled by self-association motifs and disease-associated polymorphism S704C. Biochemistry.

[48] Kamiya A., Tomoda T., Chang J., Takaki M., Zhan C., Morita M., Cascio M.B., Elashvili S., Koizumi H., Takanezawa Y. (2006). DISC1-NDEL1/NUDEL protein interaction, an essential component for neurite outgrowth, is modulated by genetic variations of DISC1. Hum. Mol. Genet..

[49] Yalla K., Elliott C., Day J.P., Findlay J., Barratt S., Hughes Z.A., Wilson L., Whiteley E., Popiolek M., Li Y. (2018). FBXW7 regulates DISC1 stability via the ubiquitin-proteosome system. Mol. Psychiatry.

[50] Mao Y., Ge X., Frank C.L., Madison J.M., Koehler A.N., Doud M.K., Tassa C., Berry E.M., Soda T., Singh K.K. (2009). Disrupted in schizophrenia 1 regulates neuronal progenitor proliferation via modulation of GSK3beta/beta-catenin signaling. Cell.

[51] Watanabe Y., Khodosevich K., Monyer H. (2014). Dendrite development regulated by the schizophrenia-associated gene FEZ1 involves the ubiquitin proteasome system. Cell Rep..

[52] Srikanth P., Lagomarsino V.N., Pearse R.V., Liao M., Ghosh S., Nehme R., Seyfried N., Eggan K., Young-Pearse T.L. (2018). Convergence of independent DISC1 mutations on impaired neurite growth via decreased UNC5D expression. Transl. Psychiatry.

[53] Salzberg Y., Pechuk V., Gat A., Setty H., Sela S., Oren-Suissa M. (2020). Synaptic Protein Degradation Controls Sexually Dimorphic Circuits through Regulation of DCC/UNC-40. Curr. Biol..

[54] Kohlbrenner E.A., Shaskan N., Pietersen C.Y., Sonntag K.C., Woo T.W. (2018). Gene expression profile associated with postnatal development of pyramidal neurons in the human prefrontal cortex implicates ubiquitin ligase E3 in the pathophysiology of schizophrenia onset. J. Psychiatr. Res..

[55] Arion D., Corradi J.P., Tang S., Datta D., Boothe F., He A., Cacace A.M., Zaczek R., Albright C.F., Tseng G. (2015). Distinctive transcriptome alterations of prefrontal pyramidal neurons in schizophrenia and schizoaffective disorder. Mol. Psychiatry.

[56] Andrews J.L., Goodfellow F.J., Matosin N., Snelling M.K., Newell K.A., Huang X.F., Fernandez-Enright F. (2017). Alterations of ubiquitin related proteins in the pathology and development of schizophrenia: Evidence from human and animal studies. J. Psychiatr. Res..

[57] Bi Y., Ren D., Yuan F., Zhang Z., Zhou D., Yi X., Ji L., Li K., Yang F., Wu X. (2023). TULP4, a novel E3 ligase gene, participates in neuronal migration as a candidate in schizophrenia. CNS Neurosci. Ther..

[58] Vawter M.P., Barrett T., Cheadle C., Sokolov B.P., Wood W.H., Donovan D.M., Webster M., Freed W.J., Becker K.G. (2001). Application of cDNA microarrays to examine gene expression differences in schizophrenia. Brain Res. Bull..

[59] Middleton F.A., Mirnics K., Pierri J.N., Lewis D.A., Levitt P. (2002). Gene expression profiling reveals alterations of specific metabolic pathways in schizophrenia. J. Neurosci..

[60] Novikova S.I., He F., Cutrufello N.J., Lidow M.S. (2006). Identification of protein biomarkers for schizophrenia and bipolar disorder in the postmortem prefrontal cortex using SELDI-TOF-MS ProteinChip profiling combined with MALDI-TOF-PSD-MS analysis. Neurobiol. Dis..

[61] Scott M.R., Rubio M.D., Haroutunian V., Meador-Woodruff J.H. (2016). Protein Expression of Proteasome Subunits in Elderly Patients with Schizophrenia. Neuropsychopharmacology.

[62] Hertzberg L., Maggio N., Muler I., Yitzhaky A., Majer M., Haroutunian V., Zuk O., Katsel P., Domany E., Weiser M. (2021). Comprehensive Gene Expression Analysis Detects Global Reduction of Proteasome Subunits in Schizophrenia. Schizophr. Bull..

[63] Bousman C.A., Luza S., Mancuso S.G., Kang D., Opazo C.M., Mostaid M.S., Cropley V., McGorry P., Shannon Weickert C., Pantelis C. (2019). Elevated ubiquitinated proteins in brain and blood of individuals with schizophrenia. Sci. Rep..

[64] Rubio M.D., Wood K., Haroutunian V., Meador-Woodruff J.H. (2013). Dysfunction of the ubiquitin proteasome and ubiquitin-like systems in schizophrenia. Neuropsychopharmacology.

[65] Scott M.R., Meador-Woodruff J.H. (2020). Intracellular compartment-specific proteasome dysfunction in postmortem cortex in schizophrenia subjects. Mol. Psychiatry.

[66] Chen X., Wang X., Sun C., Chen Q., O’Neill F.A., Walsh D., Fanous A., Kendler K.S. (2008). FBXL21 association with schizophrenia in Irish family and case-control samples. Am. J. Med. Genet. B Neuropsychiatr. Genet..

[67] Kato A., Rouach N., Nicoll R.A., Bredt D.S. (2005). Activity-dependent NMDA receptor degradation mediated by retrotranslocation and ubiquitination. Proc. Natl. Acad. Sci. USA.

[68] Han C., Cui K., Bi X., Wang L., Sun M., Yang L., Liu L. (2019). Association between polymorphism of the NEDD4 gene and cognitive dysfunction of schizophrenia patients in Chinese Han population. BMC Psychiatry.

[69] Lin A., Hou Q., Jarzylo L., Amato S., Gilbert J., Shang F., Man H.Y. (2011). Nedd4-mediated AMPA receptor ubiquitination regulates receptor turnover and trafficking. J. Neurochem..

[70] Cheng Y., Chen X., Zhang X.Q., Ju P.J., Wang W.D., Fang Y., Lin G.N., Cui D.H. (2024). Interaction between RNF4 and SART3 is associated with the risk of schizophrenia. Heliyon.

[71] Rosen D.R., Siddique T., Patterson D., Figlewicz D.A., Sapp P., Hentati A., Donaldson D., Goto J., O’Regan J.P., Deng H.X. (1993). Mutations in Cu/Zn superoxide dismutase gene are associated with familial amyotrophic lateral sclerosis. Nature.

[72] Arai T., Hasegawa M., Akiyama H., Ikeda K., Nonaka T., Mori H., Mann D., Tsuchiya K., Yoshida M., Hashizume Y. (2006). TDP-43 is a component of ubiquitin-positive tau-negative inclusions in frontotemporal lobar degeneration and amyotrophic lateral sclerosis. Biochem. Biophys. Res. Commun..

[73] Neumann M., Sampathu D.M., Kwong L.K., Truax A.C., Micsenyi M.C., Chou T.T., Bruce J., Schuck T., Grossman M., Clark C.M. (2006). Ubiquitinated TDP-43 in frontotemporal lobar degeneration and amyotrophic lateral sclerosis. Science.

[74] Kwiatkowski T.J., Bosco D.A., Leclerc A.L., Tamrazian E., Vanderburg C.R., Russ C., Davis A., Gilchrist J., Kasarskis E.J., Munsat T. (2009). Mutations in the FUS/TLS gene on chromosome 16 cause familial amyotrophic lateral sclerosis. Science.

[75] Vance C., Rogelj B., Hortobágyi T., De Vos K.J., Nishimura A.L., Sreedharan J., Hu X., Smith B., Ruddy D., Wright P. (2009). Mutations in FUS, an RNA processing protein, cause familial amyotrophic lateral sclerosis type 6. Science.

[76] Hoffman E.K., Wilcox H.M., Scott R.W., Siman R. (1996). Proteasome inhibition enhances the stability of mouse Cu/Zn superoxide dismutase with mutations linked to familial amyotrophic lateral sclerosis. J. Neurol. Sci..

[77] Johnston J.A., Dalton M.J., Gurney M.E., Kopito R.R. (2000). Formation of high molecular weight complexes of mutant Cu, Zn-superoxide dismutase in a mouse model for familial amyotrophic lateral sclerosis. Proc. Natl. Acad. Sci. USA.

[78] Niwa J., Ishigaki S., Hishikawa N., Yamamoto M., Doyu M., Murata S., Tanaka K., Taniguchi N., Sobue G. (2002). Dorfin ubiquitylates mutant SOD1 and prevents mutant SOD1-mediated neurotoxicity. J. Biol. Chem..

[79] Miyazaki K., Fujita T., Ozaki T., Kato C., Kurose Y., Sakamoto M., Kato S., Goto T., Itoyama Y., Aoki M. (2004). NEDL1, a novel ubiquitin-protein isopeptide ligase for dishevelled-1, targets mutant superoxide dismutase-1. J. Biol. Chem..

[80] Di Noto L., Whitson L.J., Cao X., Hart P.J., Levine R.L. (2005). Proteasomal degradation of mutant superoxide dismutases linked to amyotrophic lateral sclerosis. J. Biol. Chem..

[81] Kabuta T., Suzuki Y., Wada K. (2006). Degradation of amyotrophic lateral sclerosis-linked mutant Cu,Zn-superoxide dismutase proteins by macroautophagy and the proteasome. J. Biol. Chem..

[82] Ishigaki S., Niwa J., Ando Y., Yoshihara T., Sawada K., Doyu M., Yamamoto M., Kato K., Yotsumoto Y., Sobue G. (2002). Differentially expressed genes in sporadic amyotrophic lateral sclerosis spinal cords-screening by molecular indexing and subsequent cDNA microarray analysis. FEBS Lett..

[83] Urushitani M., Kurisu J., Tsukita K., Takahashi R. (2002). Proteasomal inhibition by misfolded mutant superoxide dismutase 1 induces selective motor neuron death in familial amyotrophic lateral sclerosis. J. Neurochem..

[84] Lee J.P., Gerin C., Bindokas V.P., Miller R., Ghadge G., Roos R.P. (2002). No correlation between aggregates of Cu/Zn superoxide dismutase and cell death in familial amyotrophic lateral sclerosis. J. Neurochem..

[85] Vlug A.S., Jaarsma D. (2004). Long term proteasome inhibition does not preferentially afflict motor neurons in organotypical spinal cord cultures. Amyotroph. Lateral Scler. Other Mot. Neuron Disord..

[86] Aquilano K., Rotilio G., Ciriolo M.R. (2003). Proteasome activation and nNOS down-regulation in neuroblastoma cells expressing a Cu,Zn superoxide dismutase mutant involved in familial ALS. J. Neurochem..

[87] Hyun D.H., Lee M., Halliwell B., Jenner P. (2003). Proteasomal inhibition causes the formation of protein aggregates containing a wide range of proteins, including nitrated proteins. J. Neurochem..

[88] Cheroni C., Marino M., Tortarolo M., Veglianese P., De Biasi S., Fontana E., Zuccarello L.V., Maynard C.J., Dantuma N.P., Bendotti C. (2009). Functional alterations of the ubiquitin-proteasome system in motor neurons of a mouse model of familial amyotrophic lateral sclerosis. Hum. Mol. Genet..

[89] Ying Z., Wang H., Fan H., Zhu X., Zhou J., Fei E., Wang G. (2009). Gp78, an ER associated E3, promotes SOD1 and ataxin-3 degradation. Hum. Mol. Genet..

[90] Dong L., Liu L., Li Y., Li W., Zhou L., Xia Q. (2022). E3 ligase Smurf1 protects against misfolded SOD1 in neuronal cells by promoting its K63 ubiquitylation and aggresome formation. Hum. Mol. Genet..

[91] Yonashiro R., Sugiura A., Miyachi M., Fukuda T., Matsushita N., Inatome R., Ogata Y., Suzuki T., Dohmae N., Yanagi S. (2009). Mitochondrial ubiquitin ligase MITOL ubiquitinates mutant SOD1 and attenuates mutant SOD1-induced reactive oxygen species generation. Mol. Biol. Cell.

[92] Hebron M.L., Lonskaya I., Sharpe K., Weerasinghe P.P., Algarzae N.K., Shekoyan A.R., Moussa C.E. (2013). Parkin ubiquitinates Tar-DNA binding protein-43 (TDP-43) and promotes its cytosolic accumulation via interaction with histone deacetylase 6 (HDAC6). J. Biol. Chem..

[93] Rafiee M.R., Rohban S., Davey K., Ule J., Luscombe N.M. (2023). RNA polymerase II-associated proteins reveal pathways affected in VCP-related amyotrophic lateral sclerosis. Brain.

[94] Lee Y.C., Huang W.C., Lin J.H., Kao T.J., Lin H.C., Lee K.H., Lin H.C., Shen C.J., Chang W.C., Huang C.C. (2018). Znf179 E3 ligase-mediated TDP-43 polyubiquitination is involved in TDP-43- ubiquitinated inclusions (UBI) (+)-related neurodegenerative pathology. J. Biomed. Sci..

[95] Watabe K., Kato Y., Sakuma M., Murata M., Niida-Kawaguchi M., Takemura T., Hanagata N., Tada M., Kakita A., Shibata N. (2020). Praja1 RING-finger E3 ubiquitin ligase suppresses neuronal cytoplasmic TDP-43 aggregate formation. Neuropathology.

[96] Hans F., Fiesel F.C., Strong J.C., Jäckel S., Rasse T.M., Geisler S., Springer W., Schulz J.B., Voigt A., Kahle P.J. (2014). UBE2E ubiquitin-conjugating enzymes and ubiquitin isopeptidase Y regulate TDP-43 protein ubiquitination. J. Biol. Chem..

[97] Ma P., Li Y., Wang H., Mao B. (2021). Haploinsufficiency of the TDP43 ubiquitin E3 ligase RNF220 leads to ALS-like motor neuron defects in the mouse. J. Mol. Cell Biol..

[98] Wang I.F., Wu L.S., Shen C.K. (2008). TDP-43: An emerging new player in neurodegenerative diseases. Trends Mol. Med..

[99] Buratti E., Baralle F.E. (2009). The molecular links between TDP-43 dysfunction and neurodegeneration. Adv. Genet..

[100] Farrawell N.E., McAlary L., Lum J.S., Chisholm C.G., Warraich S.T., Blair I.P., Vine K.L., Saunders D.N., Yerbury J.J. (2020). Ubiquitin Homeostasis Is Disrupted in TDP-43 and FUS Cell Models of ALS. iScience.

[101] The Huntington’s Disease Collaborative Research Group (1993). A novel gene containing a trinucleotide repeat that is expanded and unstable on Huntington’s disease chromosomes. Cell.

[102] Bence N.F., Sampat R.M., Kopito R.R. (2001). Impairment of the ubiquitin-proteasome system by protein aggregation. Science.

[103] Bennett E.J., Shaler T.A., Woodman B., Ryu K.Y., Zaitseva T.S., Becker C.H., Bates G.P., Schulman H., Kopito R.R. (2007). Global changes to the ubiquitin system in Huntington’s disease. Nature.

[104] Bhat K.P., Yan S., Wang C.E., Li S., Li X.J. (2014). Differential ubiquitination and degradation of huntingtin fragments modulated by ubiquitin-protein ligase E3A. Proc. Natl. Acad. Sci. USA.

[105] Maheshwari M., Shekhar S., Singh B.K., Jamal I., Vatsa N., Kumar V., Sharma A., Jana N.R. (2014). Deficiency of Ube3a in Huntington’s disease mice brain increases aggregate load and accelerates disease pathology. Hum. Mol. Genet..

[106] Mishra A., Dikshit P., Purkayastha S., Sharma J., Nukina N., Jana N.R. (2008). E6-AP promotes misfolded polyglutamine proteins for proteasomal degradation and suppresses polyglutamine protein aggregation and toxicity. J. Biol. Chem..

[107] Lee J.M., Wheeler V.C., Chao M.J., Vonsattel J.P.G., Pinto R.M., Lucente D., Abu-Elneel K., Ramos E.M., Mysore J.S., Gillis T. (2015). Identification of Genetic Factors that Modify Clinical Onset of Huntington’s Disease. Cell.

[108] Koyuncu S., Saez I., Lee H.J., Gutierrez-Garcia R., Pokrzywa W., Fatima A., Hoppe T., Vilchez D. (2018). The ubiquitin ligase UBR5 suppresses proteostasis collapse in pluripotent stem cells from Huntington’s disease patients. Nat. Commun..

[109] Yang H., Zhong X., Ballar P., Luo S., Shen Y., Rubinsztein D.C., Monteiro M.J., Fang S. (2007). Ubiquitin ligase Hrd1 enhances the degradation and suppresses the toxicity of polyglutamine-expanded huntingtin. Exp. Cell. Res..

[110] Polymeropoulos M.H., Lavedan C., Leroy E., Ide S.E., Dehejia A., Dutra A., Pike B., Root H., Rubenstein J., Boyer R. (1997). Mutation in the alpha-synuclein gene identified in families with Parkinson’s disease. Science.

[111] Kitada T., Asakawa S., Hattori N., Matsumine H., Yamamura Y., Minoshima S., Yokochi M., Mizuno Y., Shimizu N. (1998). Mutations in the parkin gene cause autosomal recessive juvenile parkinsonism. Nature.

[112] Bonifati V., Rizzu P., van Baren M.J., Schaap O., Breedveld G.J., Krieger E., Dekker M.C., Squitieri F., Ibanez P., Joosse M. (2003). Mutations in the DJ-1 gene associated with autosomal recessive early-onset parkinsonism. Science.

[113] Paisán-Ruíz C., Jain S., Evans E.W., Gilks W.P., Simón J., van der Brug M., López de Munain A., Aparicio S., Gil A.M., Khan N. (2004). Cloning of the gene containing mutations that cause PARK8-linked Parkinson’s disease. Neuron.

[114] Valente E.M., Abou-Sleiman P.M., Caputo V., Muqit M.M., Harvey K., Gispert S., Ali Z., Del Turco D., Bentivoglio A.R., Healy D.G. (2004). Hereditary early-onset Parkinson’s disease caused by mutations in PINK1. Science.

[115] Ramirez A., Heimbach A., Gründemann J., Stiller B., Hampshire D., Cid L.P., Goebel I., Mubaidin A.F., Wriekat A.L., Roeper J. (2006). Hereditary parkinsonism with dementia is caused by mutations in ATP13A2, encoding a lysosomal type 5 P-type ATPase. Nat. Genet..

[116] Hasegawa M., Fujiwara H., Nonaka T., Wakabayashi K., Takahashi H., Lee V.M., Trojanowski J.Q., Mann D., Iwatsubo T. (2002). Phosphorylated alpha-synuclein is ubiquitinated in alpha-synucleinopathy lesions. J. Biol. Chem..

[117] Upadhya S.C., Hegde A.N. (2003). A potential proteasome-interacting motif within the ubiquitin-like domain of parkin and other proteins. Trends Biochem. Sci..

[118] Shinbo Y., Niki T., Taira T., Ooe H., Takahashi-Niki K., Maita C., Seino C., Iguchi-Ariga S.M., Ariga H. (2006). Proper SUMO-1 conjugation is essential to DJ-1 to exert its full activities. Cell Death Differ..

[119] Ding X., Goldberg M.S. (2009). Regulation of LRRK2 stability by the E3 ubiquitin ligase CHIP. PLoS ONE.

[120] Rudenko I.N., Kaganovich A., Langston R.G., Beilina A., Ndukwe K., Kumaran R., Dillman A.A., Chia R., Cookson M.R. (2017). The G2385R risk factor for Parkinson’s disease enhances CHIP-dependent intracellular degradation of LRRK2. Biochem. J..

[121] Lee Y., Stevens D.A., Kang S.U., Jiang H., Lee Y.I., Ko H.S., Scarffe L.A., Umanah G.E., Kang H., Ham S. (2017). PINK1 Primes Parkin-Mediated Ubiquitination of PARIS in Dopaminergic Neuronal Survival. Cell Rep..

[122] Si J., Van den Haute C., Lobbestael E., Martin S., van Veen S., Vangheluwe P., Baekelandt V. (2021). ATP13A2 Regulates Cellular α-Synuclein Multimerization, Membrane Association, and Externalization. Int. J. Mol. Sci..

[123] Leroy E., Boyer R., Auburger G., Leube B., Ulm G., Mezey E., Harta G., Brownstein M.J., Jonnalagada S., Chernova T. (1998). The ubiquitin pathway in Parkinson’s disease. Nature.

[124] Kumar R., Jangir D.K., Verma G., Shekhar S., Hanpude P., Kumar S., Kumari R., Singh N., Sarovar Bhavesh N., Ranjan Jana N. (2017). S-nitrosylation of UCHL1 induces its structural instability and promotes α-synuclein aggregation. Sci. Rep..

[125] Andersson F.I., Werrell E.F., McMorran L., Crone W.J., Das C., Hsu S.T., Jackson S.E. (2011). The effect of Parkinson’s-disease-associated mutations on the deubiquitinating enzyme UCH-L1. J. Mol. Biol..

[126] Lim K.L., Dawson V.L., Dawson T.M. (2006). Parkin-mediated lysine 63-linked polyubiquitination: A link to protein inclusions formation in Parkinson’s and other conformational diseases?. Neurobiol. Aging.

[127] Olzmann J.A., Chin L.S. (2008). Parkin-mediated K63-linked polyubiquitination: A signal for targeting misfolded proteins to the aggresome-autophagy pathway. Autophagy.

[128] Chin L.S., Olzmann J.A., Li L. (2010). Parkin-mediated ubiquitin signalling in aggresome formation and autophagy. Biochem. Soc. Trans..

[129] Ordureau A., Sarraf S.A., Duda D.M., Heo J.M., Jedrychowski M.P., Sviderskiy V.O., Olszewski J.L., Koerber J.T., Xie T., Beausoleil S.A. (2014). Quantitative proteomics reveal a feedforward mechanism for mitochondrial PARKIN translocation and ubiquitin chain synthesis. Mol. Cell.

[130] Ordureau A., Heo J.M., Duda D.M., Paulo J.A., Olszewski J.L., Yanishevski D., Rinehart J., Schulman B.A., Harper J.W. (2015). Defining roles of PARKIN and ubiquitin phosphorylation by PINK1 in mitochondrial quality control using a ubiquitin replacement strategy. Proc. Natl. Acad. Sci. USA.

[131] Tsai Y.C., Fishman P.S., Thakor N.V., Oyler G.A. (2003). Parkin facilitates the elimination of expanded polyglutamine proteins and leads to preservation of proteasome function. J. Biol. Chem..

[132] Morishima Y., Wang A.M., Yu Z., Pratt W.B., Osawa Y., Lieberman A.P. (2008). CHIP deletion reveals functional redundancy of E3 ligases in promoting degradation of both signaling proteins and expanded glutamine proteins. Hum. Mol. Genet..

[133] Winborn B.J., Travis S.M., Todi S.V., Scaglione K.M., Xu P., Williams A.J., Cohen R.E., Peng J., Paulson H.L. (2008). The deubiquitinating enzyme ataxin-3, a polyglutamine disease protein, edits Lys63 linkages in mixed linkage ubiquitin chains. J. Biol. Chem..

[134] Todi S.V., Winborn B.J., Scaglione K.M., Blount J.R., Travis S.M., Paulson H.L. (2009). Ubiquitination directly enhances activity of the deubiquitinating enzyme ataxin-3. EMBO J..

[135] Olzmann J.A., Li L., Chin L.S. (2008). Aggresome formation and neurodegenerative diseases: Therapeutic implications. Curr. Med. Chem..

[136] Buhmann C., Bussopulos A., Oechsner M. (2003). Dopaminergic response in Parkinsonian phenotype of Machado-Joseph disease. Mov. Disord..

[137] Bedford L., Hay D., Devoy A., Paine S., Powe D.G., Seth R., Gray T., Topham I., Fone K., Rezvani N. (2008). Depletion of 26S proteasomes in mouse brain neurons causes neurodegeneration and Lewy-like inclusions resembling human pale bodies. J. Neurosci..

[138] Wahl C., Kautzmann S., Krebiehl G., Strauss K., Woitalla D., Müller T., Bauer P., Riess O., Krüger R. (2008). A comprehensive genetic study of the proteasomal subunit S6 ATPase in German Parkinson’s disease patients. J. Neural. Transm..

[139] Fernández-Cruz I., Sánchez-Díaz I., Narváez-Padilla V., Reynaud E. (2020). Rpt2 proteasome subunit reduction causes Parkinson’s disease like symptoms in Drosophila. IBRO Rep..

[140] Ichikawa Y., Goto J., Hattori M., Toyoda A., Ishii K., Jeong S.Y., Hashida H., Masuda N., Ogata K., Kasai F. (2001). The genomic structure and expression of MJD, the Machado-Joseph disease gene. J. Hum. Genet..

[141] Schmitt I., Linden M., Khazneh H., Evert B.O., Breuer P., Klockgether T., Wuellner U. (2007). Inactivation of the mouse Atxn3 (ataxin-3) gene increases protein ubiquitination. Biochem. Biophys. Res. Commun..

[142] Zhong X., Pittman R.N. (2006). Ataxin-3 binds VCP/p97 and regulates retrotranslocation of ERAD substrates. Hum. Mol. Genet..

[143] Ristic G., Sutton J.R., Libohova K., Todi S.V. (2018). Toxicity and aggregation of the polyglutamine disease protein, ataxin-3 is regulated by its binding to VCP/p97 in Drosophila melanogaster. Neurobiol. Dis..

[144] Wilson S.M., Bhattacharyya B., Rachel R.A., Coppola V., Tessarollo L., Householder D.B., Fletcher C.F., Miller R.J., Copeland N.G., Jenkins N.A. (2002). Synaptic defects in ataxia mice result from a mutation in Usp14, encoding a ubiquitin-specific protease. Nat. Genet..

[145] Anderson C., Crimmins S., Wilson J.A., Korbel G.A., Ploegh H.L., Wilson S.M. (2005). Loss of Usp14 results in reduced levels of ubiquitin in ataxia mice. J. Neurochem..

[146] Crimmins S., Jin Y., Wheeler C., Huffman A.K., Chapman C., Dobrunz L.E., Levey A., Roth K.A., Wilson J.A., Wilson S.M. (2006). Transgenic rescue of ataxia mice with neuronal-specific expression of ubiquitin-specific protease 14. J. Neurosci..

[147] Chen P.C., Qin L.N., Li X.M., Walters B.J., Wilson J.A., Mei L., Wilson S.M. (2009). The proteasome-associated deubiquitinating enzyme Usp14 is essential for the maintenance of synaptic ubiquitin levels and the development of neuromuscular junctions. J. Neurosci..

